# *Lacticaseibacillus rhamnosus* Strain GG (LGG) Regulate Gut Microbial Metabolites, an In Vitro Study Using Three Mature Human Gut Microbial Cultures in a Simulator of Human Intestinal Microbial Ecosystem (SHIME)

**DOI:** 10.3390/foods12112105

**Published:** 2023-05-24

**Authors:** LinShu Liu, Adrienne B. Narrowe, Jenni A. Firrman, Karley K. Mahalak, Jamshed T. Bobokalonov, Johanna M. S. Lemons, Kyle Bittinger, Scott Daniel, Ceylan Tanes, Lisa Mattei, Elliot S. Friedman, Jason W. Soares, Masuko Kobori, Wei-Bin Zeng, Peggy M. Tomasula

**Affiliations:** 1 Dairy and Functional Foods Research Unit, Eastern Regional Research Center, Agricultural Research Service, United States Department of Agriculture, Wyndmoor, PA 19038, USA; 2 V.I. Nikitin Chemistry Institute of Tajikistan Academy of Sciences, Dushanbe 734063, Tajikistan; 3 Division of Gastroenterology, Hepatology, and Nutrition, The Children’s Hospital of Philadelphia, Philadelphia, PA 19104, USA; 4 Division of Gastroenterology & Hepatology, Perelman School of Medicine, University of Pennsylvania, Philadelphia, PA 19104, USA; 5 Bioprocessing and Bioengineering Group, US Army Combat Capabilities Development Command Soldier Center (CCDC-SC), Natick, MA 01760, USA; 6 Food Research Institute, National Agriculture and Food Research Organization, Tsukuba 305-8642, Ibaraki, Japan; 7 Department of Mathematics, University of Louisville, Louisville, KY 40292, USA

**Keywords:** *Laticaseibacillus rhamnosus* strain GG, LGG, SHIME^®^, metabolome, gut microbiome, indole propionic acid

## Abstract

In the present research, we investigated changes in the gut metabolome that occurred in response to the administration of the *Laticaseibacillus rhamnosus* strain GG (LGG). The probiotics were added to the ascending colon region of mature microbial communities established in a human intestinal microbial ecosystem simulator. Shotgun metagenomic sequencing and metabolome analysis suggested that the changes in microbial community composition corresponded with changes to metabolic output, and we can infer linkages between some metabolites and microorganisms. The in vitro method permits a spatially-resolved view of metabolic transformations under human physiological conditions. By this method, we found that tryptophan and tyrosine were mainly produced in the ascending colon region, while their derivatives were detected in the transverse and descending regions, revealing sequential amino acid metabolic pathways along with the colonic tract. The addition of LGG appeared to promote the production of indole propionic acid, which is positively associated with human health. Furthermore, the microbial community responsible for the production of indole propionic acid may be broader than is currently known.

## 1. Introduction

Gut microbial metabolites are a collection of low molecular weight chemical compounds, such as amino acids, oligopeptides, fatty acids, and vitamins. The gut microbial metabolites with molecular weights less than 1000 Da are the most active and often the subject of research. They mediate the impact of the gut microbiome on host epithelial and immune cells, both locally and systemically. Such metabolites have broad functions, including serving as substrates for cell structure building, energy generation, signal transduction, and as biomarkers for disease diagnosis and therapy [1,2,3,4]. Gut metabolites are produced by microorganisms residing in the gastrointestinal tract (GIT) through the fermentation of dietary components and digestion of endogenous intestinal mucus. Diet, individual health characteristics, and environment shape the composition and ratio of gut metabolites (i.e., Western diet vs. Mediterranean diet, vegetarian vs. omnivores, climate). Due to the potential beneficial effects these gut microbial metabolites may have on host health, members of the gut microbiome have been sought out for use as probiotics.

*Lacticaseibacillus rhamnosus* strain GG (LGG) is among the most studied probiotics and is generally accepted as a food supplement globally [5]. It is also considered effective for therapeutic purposes in some countries in Asia and Europe [6]. LGG is proposed to benefit humans in several ways, including killing or inhibiting pathogens [7,8], enhancing epithelial barrier function, and modulating host immune response [9]. LGG can affect host immune responses and promote intestinal epithelial homeostasis by releasing two proteins, P40 and P75 [10]. These two soluble proteins, harvested and purified from LGG culture broth, were found to be highly effective in the prevention of cytokine-induced intestinal epithelial damage and the reduction of hydrogen peroxide-mediated disruption of the epithelial barrier function. Furthermore, research also pointed to the influence of LGG on the metabolism of host cells and the gut microbiome, cellular growth, and energy conversion [11,12,13]. It was reported in a recent experiment using gnotobiotic mice that the addition of LGG enhanced the production of indole lactic acid, acetylated amino acids, and histamine [14].

Due to the interest in the role of LGG in health promotion, many studies were conducted using animal models of chronic disease, either transgenic or chemically induced [15,16]. Biospecimens, including fecal, blood, and tissue samples, were collected after the animals were euthanized. However, what occurs at specific gastrointestinal regions post-LGG ingestion, and the timeframe of these effects, is difficult to discern using animal models, especially when data are needed frequently and repeatedly from a single subject. Probiotics, such as LGG, are generally used as a food and beverage supplement consumed regularly by healthy individuals. Studies on the effect of LGG using only disease models and with limited spatial and temporal sampling cannot identify the effects of LGG in healthy humans as typically consumed.

The primary goal of the present research was to explore the change in gut microbial metabolites in response to the addition of LGG under standardized conditions. The experiment is conducted using the Simulator of the Human Intestinal Microbial Ecosystem (SHIME) inoculated with fecal samples from three healthy Western-diet consumers. Shotgun metagenomic sequencing is used in conjunction with 16S rRNA amplicon sequencing and paired with metabolite analysis using ultra-performance liquid chromatography (UPLC), mass spectrometer (MS), and hydrophilic interaction chromatography (HILIC). Here, we describe tandem shifts in microbial community composition and metabolite profiles along regions of the simulated human colon, providing spatial resolution to key metabolic transformations and suggesting additions to the list of taxa capable of these transformations.

## 2. Materials and Methods

### 2.1. Materials

Unless otherwise indicated, all the chemical and biological reagents used in the present study were purchased from Millipore-Sigma (Saint Louis, MO, USA). Deionized water was prepared using MilliQ Water Systems OM-154 (Millipore Corporation, Bedford, MA, USA).

*Lacticaseibacillus rhamnosus* GG cells (LGG, ATCC 53103) were originally obtained from American Type Culture Collection (Manassas, VA, USA). Prior to inoculation, the cells were cultured and twice grown in de Man, Rogosa and Sharpe (MRS) broth (Becton-Dickinson (BD; Franklin Lakes, NJ, USA) anaerobically overnight at 37 °C. A fraction of the overnight culture was added to 1 L of MRS broth and grown anaerobically to mid-exponential phase by shaking at 125 rpm for 10 h at 37 °C. The culture was then centrifuged for 10 min at 4 °C at 5000× *g*. The pellet was washed with saline (0.9% NaCl) and resuspended in 25 mL of saline.

Defined Medium (DM) containing various carbohydrates, lipids, proteins, vitamins, and minerals (Table S1) for bacterial culturing in vitro was purchased from ProDigest (Gent, Belgium). Before use, the DM was dispersed in MilliQ water (14.6 g/L) under stirring, adjusted to pH 2 using HCL, and autoclaved. Pancreatic juice was prepared by dissolving 6 g/L bile salts (BD, Franklin Lakes, NJ, USA) and 0.9 g/L pancreatin in autoclaved NaHCO_3_ solutions and was stored at 4 °C until use. Porous mucin carriers were prepared by coating the microporous plastic carriers (ProDigest) with a colloidal solution of 1% bacterial agar and 5% of type II porcine mucin in autoclaved MilliQ water. The coatings were solidified under laminar flow in a biosafety cabinet at room temperature and stored at 4 °C.

Fecal homogenates were obtained from OpenBiome (Cambridge, MA, USA). According to the provider, fecal samples were collected from subjects aged 21–45 years old, Western diet consumers with average BMI and antibiotic free for a year or more. The fecal materials were mixed with glycerol buffer solution to make a 10% homogenate and then stored at −80 °C until inoculation. Immediately prior to use, the fecal material was thawed at 4 °C according to the manufacturer’s specifications.

### 2.2. SHIME Set Up and Operation

The SHIME (ProDigest; Ghent, Belgium) is a computer-controlled artificial gastrointestinal apparatus; it was set up as reported previously [17] for evaluating the impact of LGG on the change in the gut microbial metabolites.

Briefly, fecal homogenate was added into three colon bioreactors representing the ascending colon (AC), transverse colon (TC), and descending colon (DC) at 5% of the reactor volume. The apparatus was maintained at 37 °C by a water jacket and sustained anaerobically by nitrogen flow from the stomach (ST) to the small intestine (SI) to the colon. A mucosal phase and a luminal phase were configured into the three large intestine regions. The pH values for each region were controlled automatically in response to the changes in the luminal phase in the following ranges: 1.9–2.1 for ST, for SI, 5.6–5.9 for AC, 6.15–6.4 for TC, and 6.6–6.9 for DC. The present experiment was repeated three times, using fecal homogenates from three donors (representing three biological replicates, named BR1, BR2, and BR3, respectively). The SHIME was provided with fresh DM suspension 16 h after inoculation, then fed three times a day, every 8 h, with 140 mL feed suspension and 60 mL pancreatic juice. As the fluid was transported from the SI to the AC, the same volume flowed from the AC to the TC, TC to the DC, and then from DC out to waste. The volume of each colon region was constantly maintained at 500 mL (AC), 800 mL (TC), and 600 mL (DC) to keep feed retention time mimicking the profile of gastrointestinal motility. After 21–23 days of operation, 15 min prior to the first feeding of the day, a fraction of LGG suspension containing ~2.5–5 × 10^11^ CFU cells was added to each AC region, resulting in ~2.5–5 × 10^8^ CFU/mL LGG in each bioreactor. LGG was added only once for this experiment. Following the LGG administration, the community was monitored for a period of 9 days. Sampling was performed twice a week. For each time, 60 min prior to the first feeding cycle of the day, 10 mL suspension solution from the luminal phase and 25 mucin carriers were removed and replaced (Figure S1). The suspension was separated into bacteria pellet (BP) and supernatant by centrifugation at 5000× *g* for 10 min at 4 °C. The supernatant was filtered through a 0.22 μm PES filter to obtain bacteria-free supernatant (BFS). Both BP and BFS were stored at −80 °C for DNA extraction and metabolite analysis, respectively. The present research on the metabolites was performed on luminal samples.

### 2.3. Metabolite Analysis

The metabolomics analysis was contracted to Metabolon Inc. (Durham, NC, USA). A total of 48 BFS samples from the 3 BRs were analyzed and included the mature gut microbial communities prior to LGG addition up to the end of the experiment. Samples were extracted and separated as hydrophilic and hydrophobic phases. Each extract was analyzed a minimum of 4 times, using a different and independent platform each time to gain the maximum amount of data; two separate reverse phases ultra-high performance liquid chromatography (RP/UPLC-MS/MS) associated with tandem mass spectroscopy with either positive or negative ion mode electrospray ionization, as well as hydrophilic-interaction chromatography (HILIC/UPLC-MS/MS) The scan range used for these analyses covered at least 70–1000 *m*/*z*, the mass accuracy matching threshold < 10 ppm.

### 2.4. DNA Extraction and Sequencing

DNA extraction and subsequent 16S rRNA sequencing analysis and shotgun metagenomic sequencing were conducted by the CHOP Microbiome Center (Philadelphia, PA, USA) as described previously [18].

Briefly, for DNA extraction, DNA was extracted from BP using the PowerSoil DNA extraction kit (Qiagen; ThermoFisher Scientific, Waltham, MA, USA) following the provider’s manual; obtained DNA was quantified with a Quanti-iT PicoGreen Assay Kit (Invitrogen, Carlsbad, CA, USA). For 16S rRNA gene sequencing, the libraries were generated from DNA extracts using barcoded PCR primers targeting the V1-V2 region of the bacterial 16S rRNA gene [19,20,21]. The amplicons thus obtained were then sequenced on an Illumina MiSeq using a 2 × 250 bp reagent kit following the manufacturer’s guidelines (San Diego, CA, USA). DNA-free water and extraction blanks were used as negative controls, and 16S gene fragments of known abundance were used as a positive control [22]. Raw sequences were processed with QIIME2 version 2019.4 software [23]. After demultiplexing and denoising, the read pairs were merged to form an exact V1V2 sequence using DADA2 software [24] and the Green Genes database, version 13.8 [25]. The unique sequences were aligned using MAFFT [26]; a phylogenetic tree was built using FastTree [27] in the R environment.

For Shotgun sequencing, sequencing libraries were generated using the NexteraXT kit; (Illumina, San Diego, CA, USA), and sequencing was carried out on the HiSeq 2500 instrument using a 2 × 125 bp reagent kit (Illumina, San Diego, CA, USA). DNA-free water and extraction blanks were used as negative controls, and DNA from *Vibrio campbellii* and Lambda phage were used as positive controls [28]. The sequences were demultiplexed, processed using the Sunbeam pipeline, version 1.2 [29], and trimmed using Trimmomatic software and default parameters [30] to remove host and phiX-derived reads.

### 2.5. Peak-to-Trough Ratio Analysis

The peak-to-trough ratio (PTR) analysis was performed according to a recently developed dynamic estimator of microbial communities (DEMIC) algorithm [31], which quantitatively compares the growth dynamics of targeted microbial species in multi-sample communities based on relative distances of contigs from the replication origin. This algorithm was implemented in Perl and R. To adapt DEMIC for use with a representative genome (NCBI assembly ASM2650v1), the full sequence was split into 20 virtual contigs. Sequencing coverage values were first calculated from the read alignments of each sample to estimate growth dynamics. Thresholds for mapping length (≥50 bp by default), mapping quality (≥5 by default), and mismatch ratio (≤0.03 by default) were adopted. Only contigs with coverages larger than 0 in all sliding windows were kept for a sample, and these coverage values were log-transformed for the subsequent analyses. Based on contig coverage values and on principal component analysis (PCA) of contig coverages in multiple samples, DEMIC inferred relative distances of contigs from the replication origin. For a given contig cluster, a linear mixed-effects model (LMM) was then fitted for the coverage values calculated as the fixed effect and sample- and contig-specific random intercept. The PTR was then constructed from the effect size of the LMM.

### 2.6. Bioinformatic and Statistical Analysis

Shotgun metagenomic sequencing reads were trimmed using sickle (v. 1.3.3) [32] and read-based profiling was performed using MetaPhlAn3 (v. 3.0.14) against the mpa_v30_CHOCOPhlAn_201901 database [33]. Trimmed reads for each bioreplicate (*n* = 18 read sets) were co-assembled using Megahit (v 1.2.9) with default parameters [34]. The resulting assemblies were binned using Metabat2 (v 2.12.1) [35]. Bin quality and completeness were estimated using CheckM (v 1.1.2) [36], and taxonomy was assigned using GTDB-tk (v 1.7.0) [37] using the GTDB r202 database. The homology search for the fldABC genes was conducted using BLASTP [38] using the *Clostridium sporogenes* genes retrieved from the UniProt database (IDs: Q93AL8, Q93AL9, Q93AM0, Q93AM1, J7SHB8). Visualizations and statistical analysis were conducted in R [39] using the following packages: vegan, tidyverse, ggplot2, pheatmap, RColorBrewer, microViz, and factoextra. Multiple testing corrections for multivariate linear models and ANOVA used the Benjamini Hochberg method, with Tukey’s HSD, to identify significant pairwise comparisons from the ANOVA results.

## 3. Results and Discussions

The transition time of ingesta in the large colon of healthy humans is much longer than in the small intestine and stomach. Most probiotic activities were discovered in the large intestinal region [8,10,40,41]. We have shown that an in vitro mature gut microbial community representing the large intestine could be established in SHIME and remain stable for 5 weeks, providing a reasonable time frame for tracing the profile of ingesta in the lower colonic region [17,18,42]. In the present research, LGG cells were directly added to the AC region of the stable microbial community. We then evaluated the viability of LGG by determining its growth and replication in the gut microbial community using the DEMIC algorithm [31] and metagenomic sequencing results.

### 3.1. LGG Survival and Replication Subsection

Prior to the addition of LGG to the SHIME, *Lacticaseibacillus* sp. was not identified in metagenomic sequencing reads using MetaPhlAn3 [33]. To confirm that there was no endogenous *Lacticaseibacillus* present in the inoculum, we additionally mapped metagenomic sequencing reads to the *Lacticaseibacillus rhamnosus* GG genome using bbmap (v. 38.79) (sourceforge.net/projects/bbmap/). As seen in Figure S2, the sequence reads mapped are consistent with the MetaPhlAn3 estimated relative abundances and suggest only the presence of the single strain added. The majority of the reads mapped in ‘perfect mode’ (Figure S2 filled circles), with only a small number of reads mapping with errors (Figure S2 transparent circles) which is likely to indicate sequencing errors, but may indicate slight sequence differences from the reference genome. Having ascertained that the only *Lacticaseibacillus* in the system was the experimentally added strain, we next estimated the replication rate of LGG in the SHIME reactors using DEMIC.

**Figure 1:**
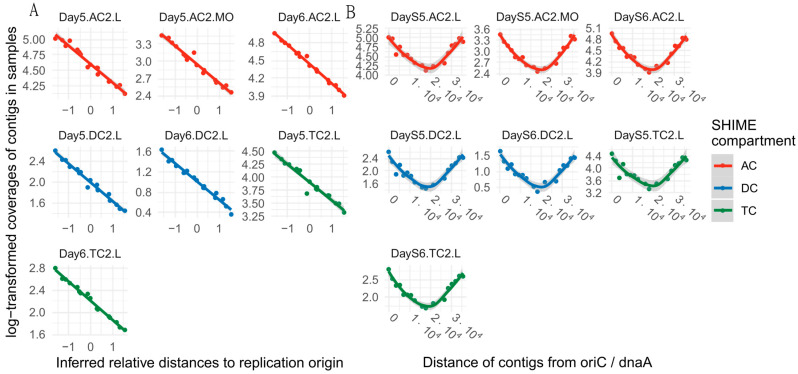
Analysis of LGG replication in SHIME by dynamic estimator of microbial communities (DEMIC) algorithm. (**A**) Inferred relative distances to replication origin, (**B**) Distance of Contigs from OriC/dnaA. The shaded areas indicate a 99% confidence interval.

Since the single circular chromosome of LGG replicates from a fixed origin to a fixed terminus bidirectionally, the growth rate can be estimated from read coverage. By leveraging multiple metagenomic sampling of bacterial communities using shotgun sequencing, DEMIC estimates bacterial growth rates of taxa within those samples by inferring relative distances of contigs from the origin of replication [31]. A linear regression of log-transformed coverage to contigs (Figure 1A) was used to estimate the PTR of the samples derived from BR2 on days 5 and 6. The LGG at both time points showed PTR values greater than one, indicating highly dynamic replication activity (Figure 1B) and confirming the growth of LGG.

### 3.2. Shotgun Metagenomic Sequencing

In this study, the SHIME platform was applied to evaluate the impact of LGG on the metabolism of the resident gut microbial community. The SHIME allowed us to monitor dynamic changes in the gut microbial community and metabolite composition in any colonic region at any designated time point. During the 9-day period from LGG addition until the end of the experiment, 180 samples were taken from the luminal and mucosal phases of the AC, TC, and DC regions for the three bioreplicates. We performed shotgun metagenomic sequencing on 18 luminal samples from each bioreplicate, yielding an average of 2,305,546 ± 537,620 reads, providing us with the opportunity to profile both the composition of the microbial community and its functional potential.

**Figure 2:**
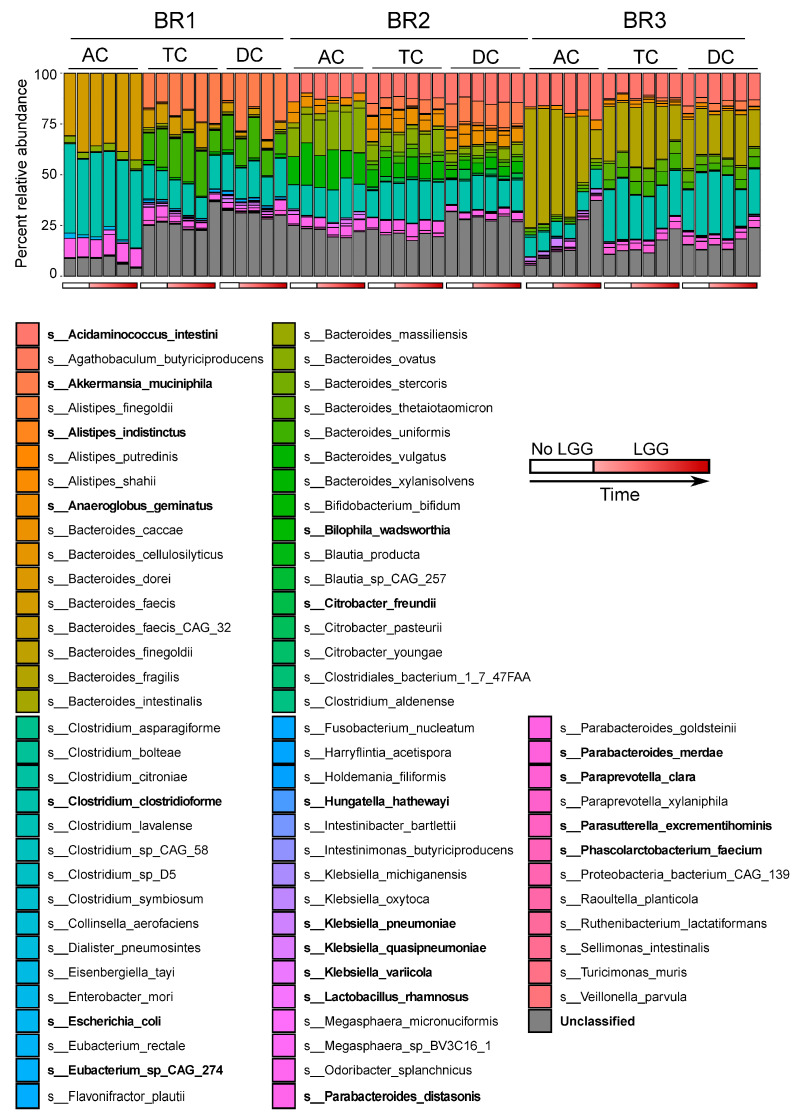
Microbial community structure inferred from metagenomic sequencing data. Relative abundances of taxa at the species level for the three bioreplicates (BR1, BR2, BR3) for three simulated colonic regions. Taxonomy was assigned to species level, with fully unclassified reads shown in grey. Taxon names in bold indicate species for which a metagenome-assembled genome (MAG) was recovered. Bars below individual columns indicate if the sample had LGG added, sampling time increases from left to right.

Metagenomic read-based taxonomic classification was implemented using MetaPhlAn3 based on shotgun sequencing. This analysis generated species-level taxonomic assignments and relative abundances (Figure 2). The communities generated from the three bioreplicates show several common characteristics. First, the bacterial compositions in the TC and DC regions are similar to each other but different from the AC region. That is consistent with our previous finding, where the bacterial community at steady state showed a strong association in composition between TC and DC and a relatively weak connection to the AC communities [17]. The inclusion of exogenous LGG does not change this trend. As only sterilized DM was added to the reactors, and the same inoculum was introduced to bioreactors representing the different colonic regions, this does indicate that the unclassified taxa were, in fact, present in the AC samples, but likely to be at lower abundances until reaching more favorable conditions in the TC and DC region reactors. Second, the compositions were donor-specific; for example, *Klebsiella* sp. was detected in the TC and DC, but not the AC of BR1; in the AC of BR2 and BR3, but not in TC and DC of these 2 BRs.

**Figure 3:**
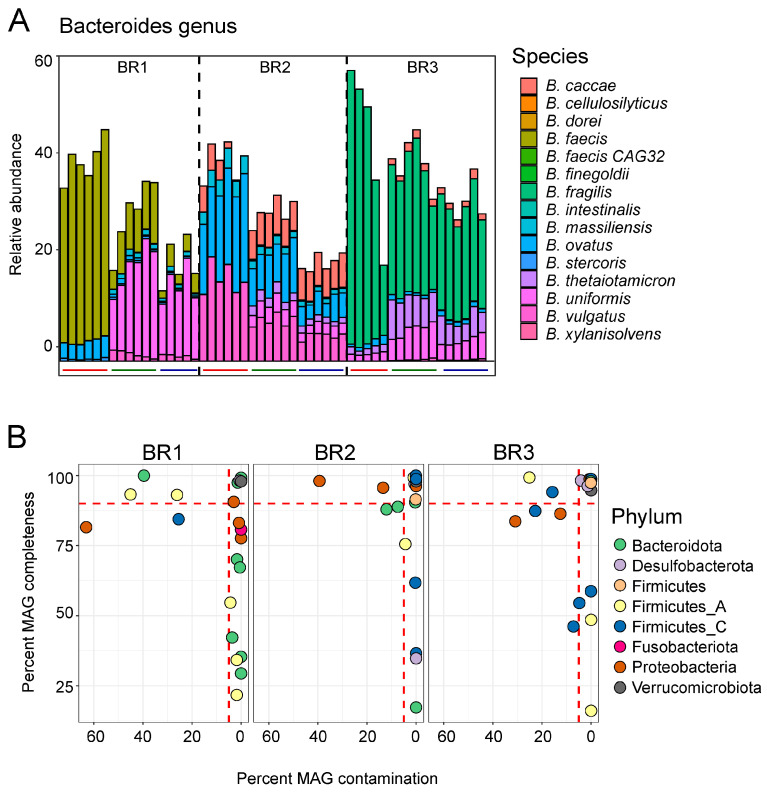
Species-level diversity within the genus Bacteroides. (**A**) A subset of metagenomic abundance data used in Figure 2, here showing only the species in the genus Bacteroides. Overall proportions are similar, while species-level assignment varies greatly. Bars below individual columns indicate reactors: red, AC; green, TC; blue, DC. (**B**) MAGs recovered from the metagenomic co-assemblies. Circles represent individual genome bins, showing estimated completeness and contamination, high-quality MAG limits are shown by red dashed lines.

Additional inter-individual differences were observed at the species level. For example, while the genus Bacteroides displayed similar distributions for each bioreplicate, accounting for a larger percentage of the community in the AC (41–45% relative abundance) than in the TC or DC (17–41% relative abundance), the species-level makeup varied greatly by individual, and in the case of BR1, shifted dramatically from AC to TC and DC (Figure 3A).

We next performed co-assembly and binning of the shotgun metagenomic samples within each bioreplicate, yielding a total of 27 medium-to-high metagenome-assembled genomes (MAGs). Of these, 21 MAGs were high quality, as indicated by MAG completeness of >90% and MAG contamination of <5% (Figure 3B). These MAGs included representatives of some of the most abundant taxa identified in 16S rRNA sequencing [43] and via read-based analysis of the metagenomes. Those species for which MAGs were assembled are shown in bold text in Figure 2.

### 3.3. Association of Microbiome and Metabolome

**Figure 4:**
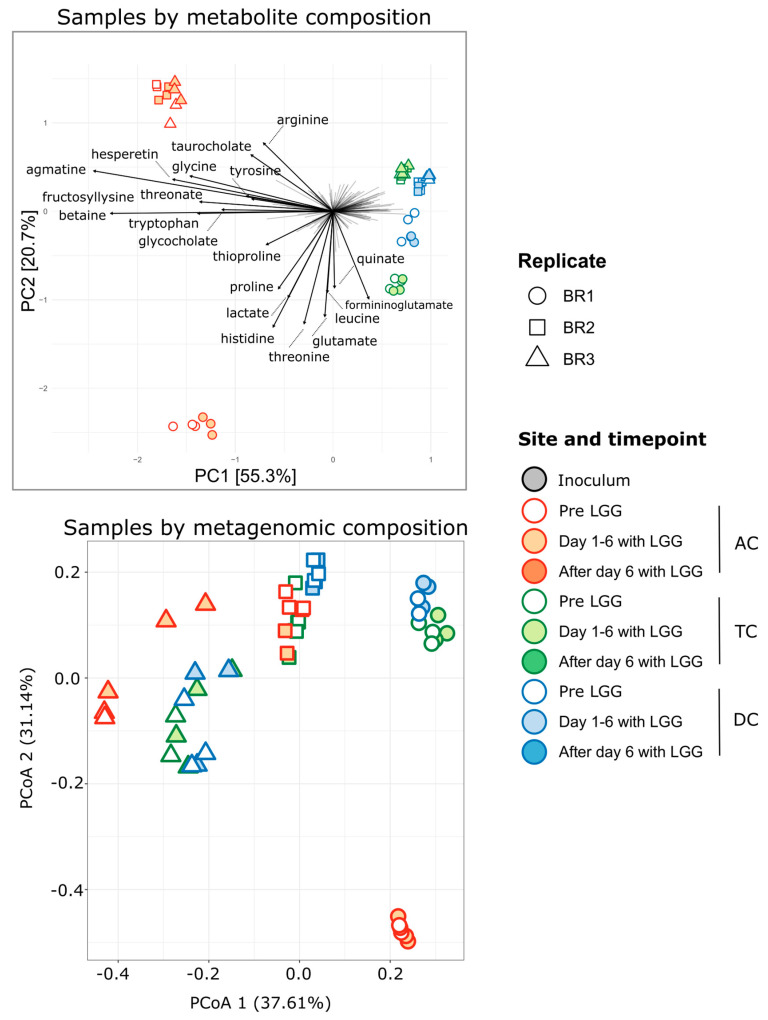
Principle components analysis of metabolite profiles (**top**) and microbial community composition (**bottom**) uncovers a similar pattern of inter-sample similarities. Genus-level relative abundances from metabolite profiles are CLR transformed prior to PCA. The bottom plot represents a PCoA of Bray–Curtis dissimilarity using genus-level relative abundances from the metagenomic data. Arrows indicate metabolite loading vectors, with the 20 metabolites providing the largest contributions labeled.

For 148 samples, we performed untargeted metabolomics yielding profiles for 654 metabolites, of which 559 could be assigned chemical identities. We first queried whether the metabolite profiles corresponded with the microbial community profiles. In a situation of functional redundancy, it is possible that the microbial community can change without producing a corresponding change in the metabolite profile. Conversely, it is possible for metabolite profiles to change without shifts in taxa, indicating changes in gene expression and metabolism by a stable community. However, in this experiment, we found that both the microbial community and the metabolite profiles shifted in concert. Principle component analysis (PCA) (Figure 4 top) of the samples based on metabolite composition agreed with Principle coordinate analysis (PCoA) performed using genus-level community profiles derived from both the shotgun metagenomic sequencing data (Figure 4 bottom) and with the samples as previously profiled using 16S rRNA amplicon data [43]. In the metabolites, we observed separation of the AC from the TC and further separation by bioreplicate, with BR2 and BR3 clustering more closely to each other than to BR1. For the metabolite profile, the TC and DC of BR2 were distinct, while for BR3, they were much more similar, whereas in the metagenomic community profile, the differences between the distal regions are less pronounced. A Mantel test of the correlation between distance matrices of samples as described by 16S rRNA ASVs and samples as described by metabolites showed significant congruence between the two profiles (Mantel statistic 0.5097 with *p*-value < 0.0001, *n* = 9999 permutations). This similarity is seen in the largely consistent groupings of samples in both the community and metabolite ordination plots (Figure 4). Taken together, these results suggested that changes in microbial community composition in the samples agreed with changes in metabolite profiles and that it may be possible to link specific metabolites to specific microorganisms.

In this context, we focused on the changes in metabolite profiles following LGG addition. As is true for the members of the microbial community, most metabolites displayed specificity for a region within the simulated colon. Individual metabolites were typically highest in either the AC or the TC and DC regions. The distinction between TC and DC was less apparent in most cases, and whether the metabolite was higher in TC or DC changed over the course of the experiment and differed by bioreplicate. However, for some metabolites (i.e., 3,4-dihydroxybutyrate), there were no clear location trends. This may have been the result of interindividual differences in the microbiome or a measurement artifact in the case of low-concentration metabolites.

However, in both cases, the difference between the LGG-treated and untreated samples was small. Some separation of samples based on metabolites was seen for the AC samples (filled vs. unfilled shapes), and a lesser degree of separation was seen among the TC samples (Figure 4, top). This suggested that if the metabolite shifts underlying this separation can be attributed to the addition of LGG to the SHIME, they occurred primarily in the AC reactors and may have been specific to the communities found there. Importantly, despite the strong inter-individual differences, this separation by treatment was found across experimental replicates. To test if the metabolite profiles and individual metabolites can distinguish these more subtle differences between LGG-treated and untreated samples, we trained a Random Forest classifier on the metabolite profiles using 70% of the data as a training set. Testing the classifier on the remaining 30% of the data, we calculated an average classification error of 9% for LGG samples and 26% for control samples, with an overall test error of 12.5% (*n* = 200 iterations). These results suggested that we can accurately classify LGG-treated samples using metabolite profiles. From the fact that the control samples are more prone to false classification than the LGG samples, we infer that there is no clear ‘untreated’ metabolome type, whereas the LGG samples present a more discernable metabolome. Interestingly, the most commonly misclassified LGG samples were from the AC communities 3 days after the addition of LGG. This suggests that the effects of LGG addition on the metabolome are short-lived.

### 3.4. Spatial Resolution of Metabolite Transformations

**Figure 5:**
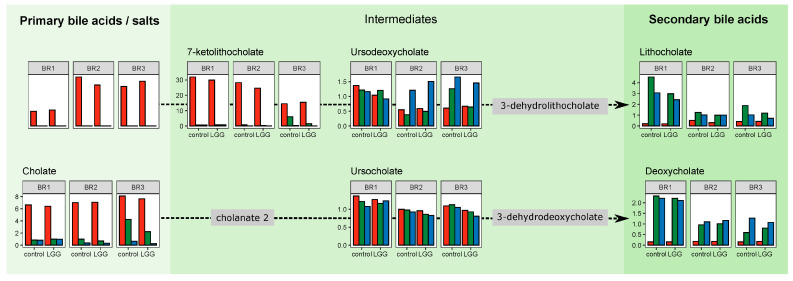
Metabolite intensity profiles for bile acids/salts and intermediates. The metabolite distributions across the colon regions track with expected microbial transformations.

The structure of the SHIME permits a spatially resolved view of metabolic transformations in a simulated human gastrointestinal tract. Intensive sampling of the lower GI tract is not feasible, the more proximal regions of the lower GI tract are less accessible for sampling, and fecal samples may not provide an accurate view of microbially mediated transformations occurring earlier in digestion. In this host-free system, we can track metabolite transformations from the ascending, through the transverse and to the descending colon. To validate these observations, we first looked at the well-known pathway of bile acid conversion. Bile acids/salts have key roles in digestion, with specific bile acids known to be produced only by the host and other derivatives known to be produced microbially. These transformations have been demonstrated in the human colon, so they are expected in this system as well. The addition of pancreatic juice to the SHIME introduces primary bile acids and salts, including cholic acid (CA), chenodeoxycholic acid (CDCA), and their taurine and glycine conjugates. In our untargeted metabolite data, we detected CA, CDCA, and two of the conjugates, taurocholic acid (TA) and glycocholic acid (GA). As shown in Figure 5, these primary bile acids/salts are found only in the AC where they were introduced, with some residual CA present in the TC and DC reactors. Tracking the intermediates 7-ketolithocholate, ursodeoxycholate, and ursocholate, we found increasing amounts of these downstream compounds in the TC and DC reactors. We detected the secondary bile acids lithocholate and deoxycholate almost exclusively in the TC and DC reactors. As these secondary bile acids are known to be the result of microbial metabolism, these results demonstrate the rapid and spatially defined transformations in the colon. These results were similar to our previous results, which found that primary bile acids present in the AC were converted to secondary bile acids in the TC and DC regions [18]. Having demonstrated that our system recapitulates the known spatial resolution of the bile acid metabolic conversions, we next looked at the spatial conversions of individual metabolites and their associations with the microbial community.

### 3.5. Individual Metabolites and Pathways

**Figure 6:**
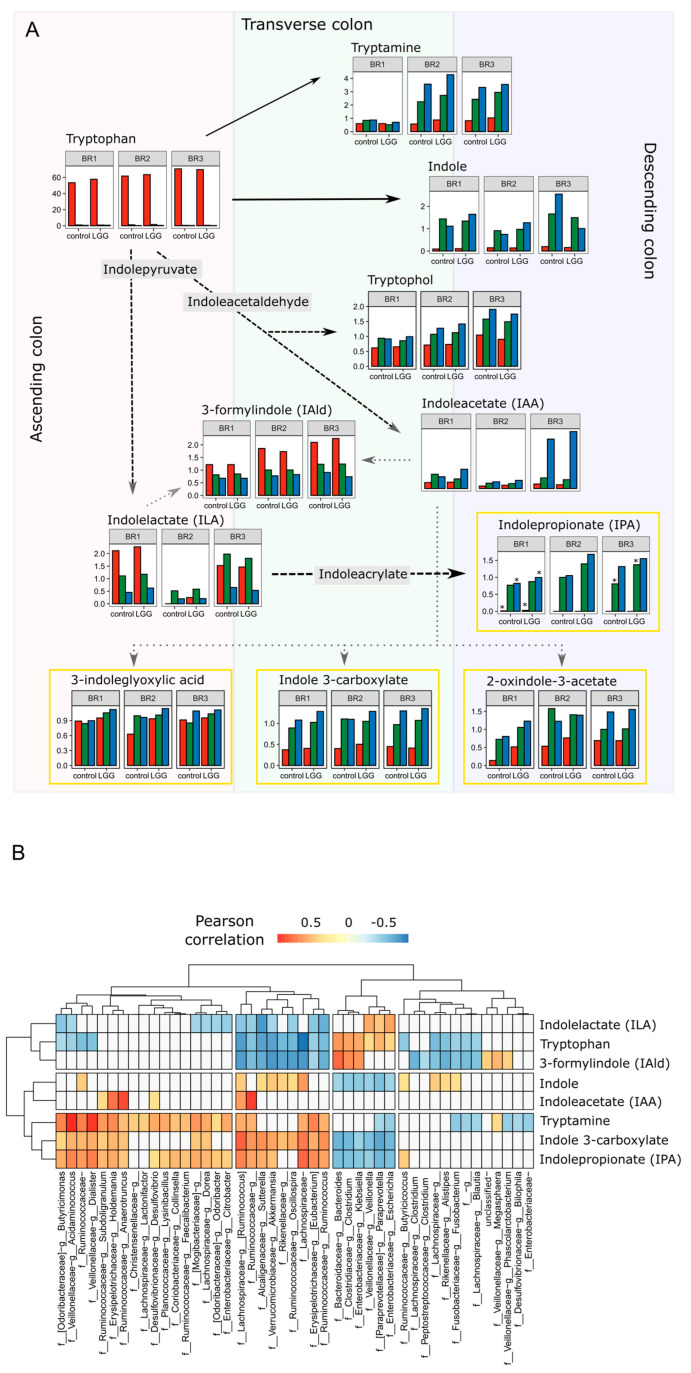
Tryptophan catabolism along the colonic gradient. (**A**) Metabolite intensity profiles for tryptophan catabolism show region-specific metabolites and suggest the location of transformations. The solid arrows indicate direct transformations and dashed arrows indicate multi-step transformations where an intermediate was not reported in the metabolite data. Those intermediates are named in grey boxes. The dotted arrows indicate multiple possible pathways. Yellow boxes surround metabolites which differ significantly by LGG treatment (BH corrected *p* < 0.05). For indolepropionate, stars indicate which regions/bioreplicates show significant differences. Metabolite profiles are superimposed on the colonic region where they are most prevalent. Arrows indicate metabolic pathways, not movement through the reactors. (**B**) Correlations of specific genus level taxa (16S rRNA profiles) with metabolites in the tryptophan pathway. The colored boxes indicate a significant Pearson correlation (BH corrected *p* < 0.05), with the color indicating the direction and magnitude of the correlation. Unfilled boxes indicate no correlation.

The most well-known of the gut microbial metabolites is the SCFA. The impact of LGG on SCFA and their roles in energy conversion and signal transduction have been discussed previously [17,43]. Going beyond SCFA, here, we identified 132 metabolites and 20 metabolic sub-pathways, which differed significantly by treatment, controlling for bioreplicate and colon region (Figure S1). While this number (132 metabolites) represents 24% of the annotated metabolites, the subtle overall differences among samples observed when described by aggregate metabolite profiles (Section 3.3 above) incorporate the influence of the remaining 76% of annotated metabolites which did not differ significantly by treatment. Among these metabolites were several indole derivatives, which are part of the tryptophan (Trp) metabolism pathway, as well as 4-hydroxyphenylacetate from the tyrosine (Tyr) metabolic pathway. Like the bile acids, these aromatic amino acids and their derivatives showed colon region-specific patterns which largely track with the catabolic pathways. The amino acid Trp, and to a lesser degree phenylalanine (Phe), and Tyr were present only in the AC region (Figure 6A, Figures S3 and S4). This restricted pattern suggests that these amino acids were produced by the proteolytic activity of the microbial community in these reactors acting on the proteins found in the feed. The near total absence of these amino acids in the more distal regions of the colon suggested that they were metabolized rapidly upon release and before reaching the TC region. Trp intensities correlated significantly with the ASV relative abundances of several taxa, including *Bacteroides*, *Klebsiella*, *Veillonella*, *Clostridium*, *Paraprevotella*, and *Escherichia sp*. (Figure 6B). Members of the *Bacteroides* are proteolytic, while members of the genera *Klebsiella* and *Escherichia* are known to produce indole from Trp. Working in concert, these taxa may be jointly producing and consuming Trp.

Downstream intermediate metabolites such as Trp, indolelactate (ILA), and indolealdehyde (3-formylindole; I3A) began to appear in the AC and continued to accumulate in the TC and DC regions. Terminal components of these pathways, such as indole and indolepropionate (IPA), appeared and accumulated only in the TC and DC regions. We observed similar transformations in the Tyr and Phe pathways, in all cases finding that the propionic acid derivatives appeared almost exclusively in the more distal regions of the colon. Unlike Trp and Tyr, Phe was found beyond the AC, suggesting it may be more recalcitrant to microbial breakdown.

Among the metabolites which changed significantly following the addition of LGG was the tryptophan derivative IPA. IPA is a gut metabolite that is of great interest due to its beneficial effects on the host through its anti-inflammatory and antioxidant properties [44,45,46]. To date, IPA is known to be produced by only six species of *Clostridium* and four species of *Peptostreptococcus* [3].

Like IPA, Phe derivatives of 3-phenylpropionate and 4-hydroxyphenyl propionate are known to be produced via a similar pathway to that yielding IPA using the fldABC gene cluster [47]. In this study, IPA (Figure 6) increased in both the TC and DC regions, further increasing over time following the LGG addition to the SHIME system. As IPA is readily absorbed by the host, it would have been difficult to localize its production and accumulation in vivo. Production of tryptophan metabolites in conjunction with shifts in the microbiome has been shown in vivo in a mouse model [28]. Here, our controlled human gut-simulation system offered the chance to understand the makeup of the community associated with the production of IPA resolved to colon-specific regions. Importantly, the propionic acid derivatives of the other aromatic amino acids also increased in the TC and DC following LGG addition to the system (Figure 6). This suggested that the presence and accumulation of IPA are microbially derived and that the genes and organisms involved in IPA synthesis from Trp catabolism were likely additionally metabolizing Phe and Tyr in the same manner.

While IPA production is most closely associated with *C. sporogenes* and other members of the genus *Clostridium* (family *Clostridiaceae*), analysis of 16S rRNA amplicon sequencing data and metagenomic sequencing data did not indicate the presence of *C. sporogenes* in any of the three bioreplicates. We did identify 16S rRNA gene sequences classified as genus *Clostridium*. However, the summed relative abundance of these sequences reached only 1.5% in a single sample and otherwise reached only 0.5% relative abundance, which did not reflect the IPA concentration profile. IPA has also been reported to be produced by the Firmicute *Peptostreptococcus anaerobius*. As with the genus *Clostridium*, in both data sets, we detected low abundances of taxa associated with the family *Peptostreptococcaceae*, almost exclusively in the DC region of BR1 and only reaching a maximum relative abundance of 1.3% in a single sample. Based on this distribution pattern, it is unlikely that members of these taxa were contributing to IPA production.

To determine which organisms might be responsible for the observed IPA and the other aromatic propionic acid metabolites, we searched the assembled metagenomic contigs for the fldABC genes known to be responsible for the production of propionic derivatives of the aromatic amino acids. In BR1, candidate fldBC genes were found to be associated with *Enterocloster*, *Fusobacterium*, and a potentially unclassified *Clostridium* species. In both BR2 and BR3, candidate fldBC genes were found to be associated with *Enterocloster*, *Acidaminococcus* and *Megasphaera*, all members of the Firmicutes. In all cases, these hits (BLASTP) were low identity members of the larger hydroxyglutaryl-CoA dehydratase family, having less than 60% amino acid identity to *C. sporogenes* fldABC genes. This lack of identity could be indicative of functional and phylogenetic distance, and experimental validation of these organisms’ or these genes’ ability to contribute to the reductive catabolic pathway of aromatic amino acid metabolism would be needed. Interestingly in both the 16S rRNA and metagenomic data, *Megasphaera* and *Acidaminococcus* sp. were found only in BR2 and BR3 bioreplicates. This suggested that if they are, in fact, producing IPA, then another taxon must be responsible for the production of this metabolite in the BR1 reactors and may be primarily responsible across all reactors, with smaller contributions by *Megasphaera* and *Acidaminococcus* sp.

Among the taxa contributing putative fldBC genes, members of the *Enterocloster* genus are very abundant in this experiment. From the metagenomic data, we successfully assembled and binned several MAGs from the genus *Enterocloster* (family *Lachnospiraceae*), including three classified as *Enterocloster clostridioformis*, one as *Enterocloster asparagiformis*, and one unclassified to species level. The GTDB database which was used to assign taxonomy to the MAGs indicates *Enterocloster clostridioformis* as the new name for *C. clostridioforme.* This taxon was formerly classified as genus *Clostridium*, family *Lachnospiraceae*. These MAGs lack 16S rRNA genes, which frequently happens in the case of metagenomic assemblies, so we cannot directly link them to the 16S rRNA data. However, MetaPhlAn3 analysis of the metagenomic sequencing reads indicated a high abundance of species *Clostridium clostridioforme*. Additionally, in the 16S rRNA amplicon data, ASVs classified as *C. clostridioforme* (i.e., *Enterocloster clostridioformis*) were also highly abundant.

Correlation analysis of the Trp metabolites with the family and genus level abundances from the 16S rRNA amplicon data showed that unclassified genera in the family *Lachnospiraceae* were the most highly correlated with IPA, while ASVs assigned to genus *Clostridium*, family *Clostridiaceae* were in fact significantly, negatively correlated with IPA. Taken together, these data suggest that members of the genus *Clostridium* (*f. Clostridiaceae*) may not be responsible for IPA production in this experiment and suggest a possible role for other taxa. While production by organisms outside the Clostridiales order is not reported [3,47], we hypothesize that these organisms could be responsible for all or part of the observed IPA production in this experiment. The small but significant association with the addition of LGG hints at a broader network of microbial interactions which may indirectly affect the production of metabolites with known effects on the host.

## 4. Conclusions

In this study, LGG was inoculated into three mature gut microbial communities cultivated in the SHIME, which includes vessels mimicking the AC, TC, and DC colon regions. Based on our previous research, we know that the composition and structure of the bacterial communities in the TC and DC regions are similar to one other but different from the AC. The addition of LGG did not interfere with this paradigm. Because we could repeatedly sample all three of these regions, we were able to monitor the kinetics and localization of metabolite production/consumption and correlate that with the presence of different bacteria and relevant gene clusters. In this way, we were able to show that the addition of the probiotic LGG promotes the production of IPA and several other tryptophan-pathway metabolites, which are positively associated with human health. While none of the known IPA producers were present at high enough levels to account for its accumulation in the TC and DC, we were able to identify other candidate organisms by mining the metagenomic data for the pertinent fldABC genes. Our analysis suggests that the microbial community capable of IPA production may be broader than is currently appreciated and provides a roadmap for the identification of unknown taxa involved in other metabolic pathways. While the magnitude of the LGG’s effect did vary between individuals, we observed similar trends in metabolite production and consumption in the different regions modeled. Our results suggest that repeated LGG administration every few days may be enough to maintain the active community and metabolome associated with LGG. These results were obtained under conditions solely excluding the interference from the mammalian milieu, which removes all bidirectional influences on the gut microbiota from the main circular system. A combinational study using both a simulator and a correlative animal model is required for a dimensional and multifaceted image of the interactions of LGG, diet, and host gut microbiota.

## Data Availability

Raw sequencing data are available in the NCBI Sequence Read Archive under BioProject accession PRJNA893635.

## Supplementary Materials

The following supporting information can be downloaded at: https://www.mdpi.com/article/10.3390/foods12112105/s1, Table S1: Composition of the defined medium; Figure S1: Schematic of SHIME system and experimental design; Figure S2: Estimates of *Lacticaseibacillus rhamnosus* GG abundance in metagenomic sequencing data; Figure S3: Schematic showing phenylalanine metabolites and pathways arranged as described for Figure 6; Figure S4: Schematic showing tyrosine metabolites and pathways arranged as described for Figure 6.
