# Supplementary material for: Lacticaseibacillus rhamnosus Strain GG (LGG) Regulate Gut Microbial Metabolites, an In Vitro Study Using Three Mature Human Gut Microbial Cultures in a Simulator of Human Intestinal Microbial Ecosystem (SHIME)
Source: Foods. 2023 May 24;12(11):2105. doi: 10.3390/foods12112105 (PMC10252382; doi:10.3390/foods12112105)
Supplement: Supplementary file 1 [file foods-12-02105-s001.zip › Figure S3.pdf]

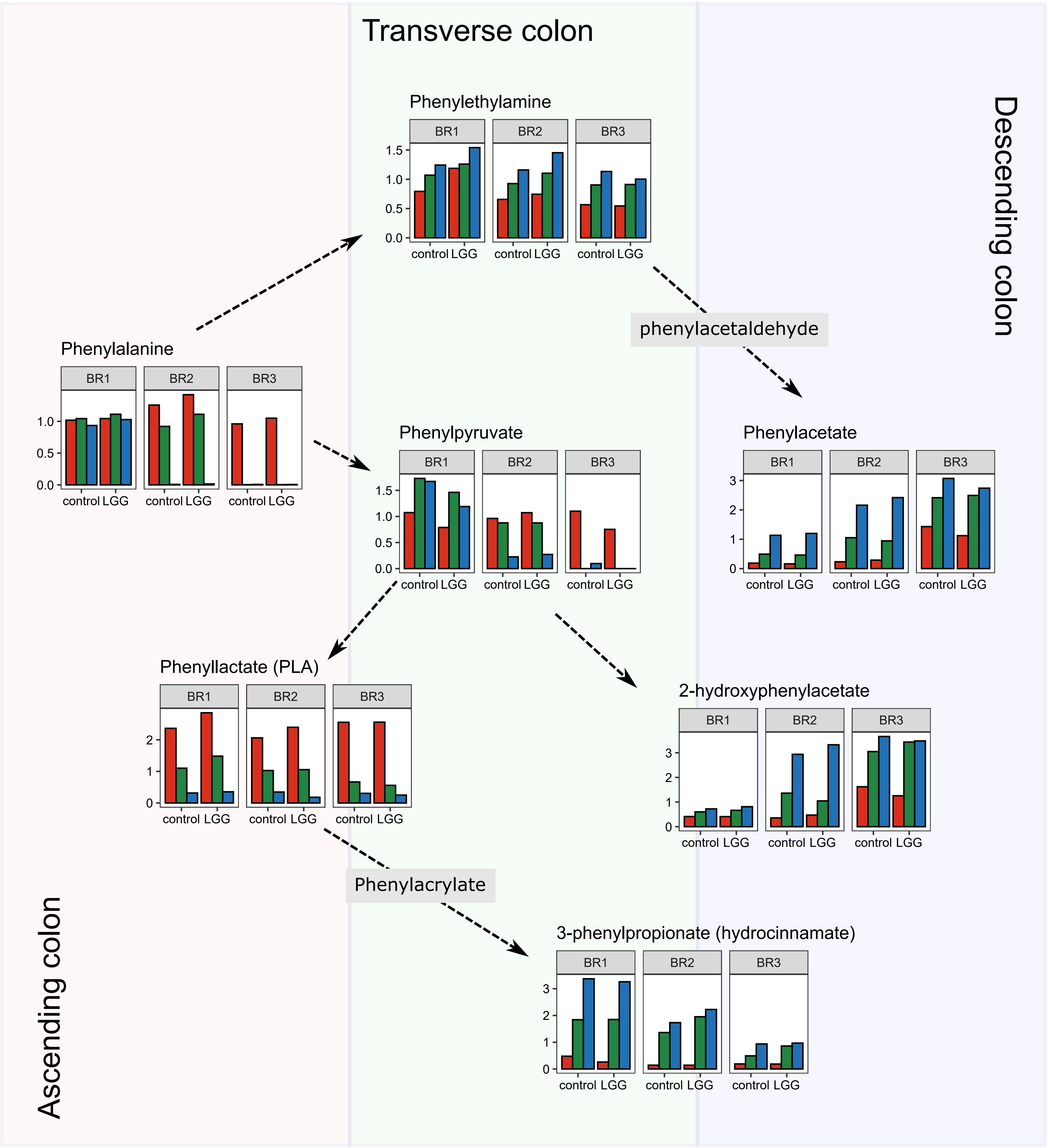
Transverse colon
Descending colon
Ascending colon
Phenylalanine
Phenylethylamine
phenylacetaldehyde
Phenylacetate
Phenylpyruvate
Phenyllactate (PLA)
2-hydroxyphenylacetate
Phenylacrylate
3-phenylpropionate (hydrocinnamate)
BR1
BR2
BR3
control
LGG
