# Supplementary material for: Lacticaseibacillus rhamnosus Strain GG (LGG) Regulate Gut Microbial Metabolites, an In Vitro Study Using Three Mature Human Gut Microbial Cultures in a Simulator of Human Intestinal Microbial Ecosystem (SHIME)
Source: Foods. 2023 May 24;12(11):2105. doi: 10.3390/foods12112105 (PMC10252382; doi:10.3390/foods12112105)
Supplement: Supplementary file 1 [file foods-12-02105-s001.zip › Figure S4.pdf]

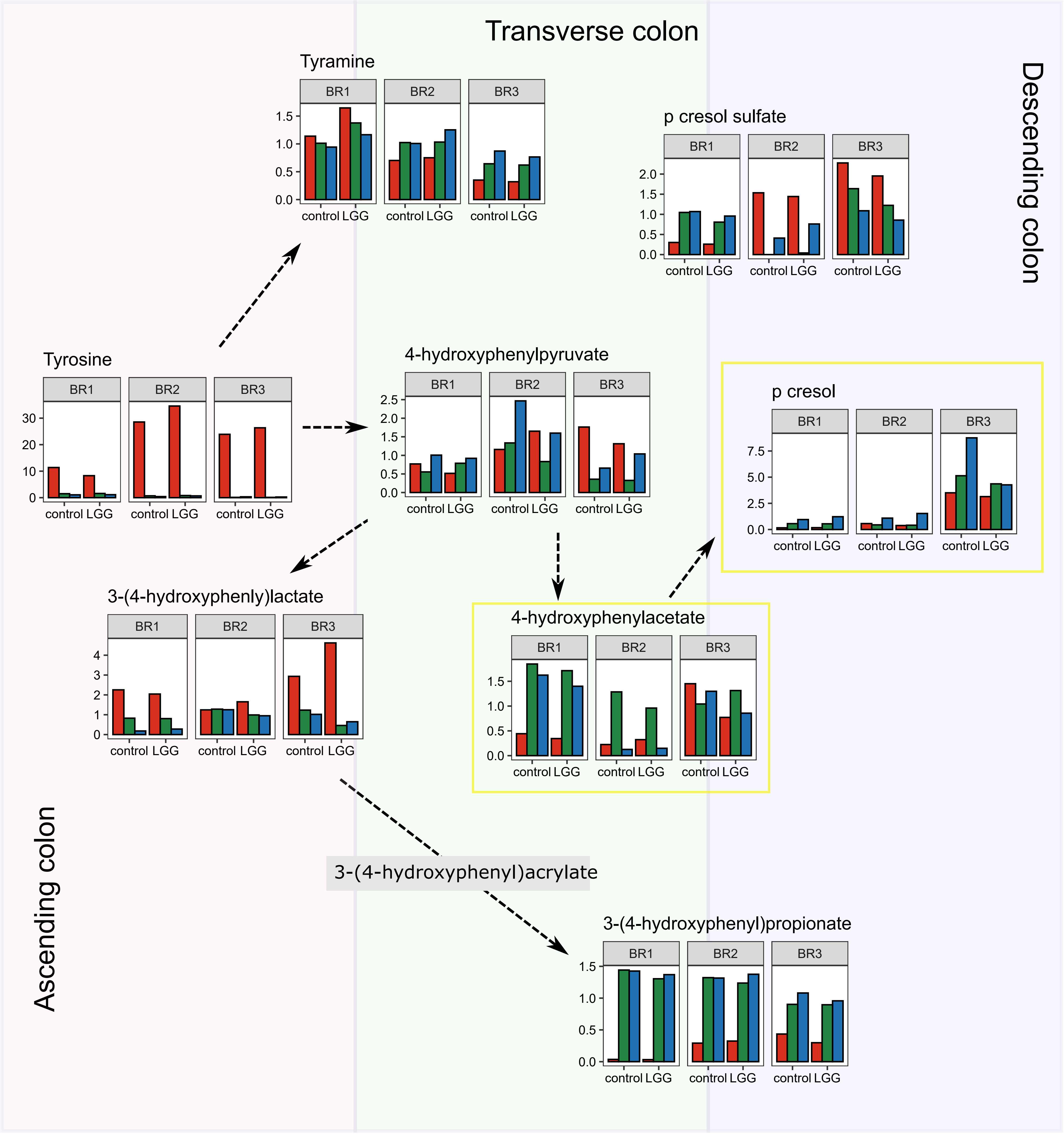

Transverse colon
Descending colon
Ascending colon
Tyramine
p cresol sulfate
Tyrosine
4-hydroxyphenylpyruvate
p cresol
3-(4-hydroxyphenly)lactate
4-hydroxyphenylacetate
3-(4-hydroxyphenyl)acrylate
3-(4-hydroxyphenyl)propionate
BR1
BR2
BR3
control LGG
