# Supplementary material for: Lacticaseibacillus rhamnosus Strain GG (LGG) Regulate Gut Microbial Metabolites, an In Vitro Study Using Three Mature Human Gut Microbial Cultures in a Simulator of Human Intestinal Microbial Ecosystem (SHIME)
Source: Foods. 2023 May 24;12(11):2105. doi: 10.3390/foods12112105 (PMC10252382; doi:10.3390/foods12112105)
Supplement: Supplementary file 1 [file foods-12-02105-s001.zip › Table S1.pdf]

Supplementary Table S1. Composition of Defined Medium

Component	Amount, g/L
Arabinogalactan	1.2
Pectin	2.0
Xylan	0.5
Glucose	0.4
Yeast Extract	3.0
Special Pepton	1.0
Mucin	2.0
L-Cystein-HCl	0.5
Starch	4.0
