# Supplementary figures and images for: Lacticaseibacillus rhamnosus Strain GG (LGG) Regulate Gut Microbial Metabolites, an In Vitro Study Using Three Mature Human Gut Microbial Cultures in a Simulator of Human Intestinal Microbial Ecosystem (SHIME)
Source: Foods. 2023 May 24;12(11):2105. doi: 10.3390/foods12112105 (PMC10252382; doi:10.3390/foods12112105)

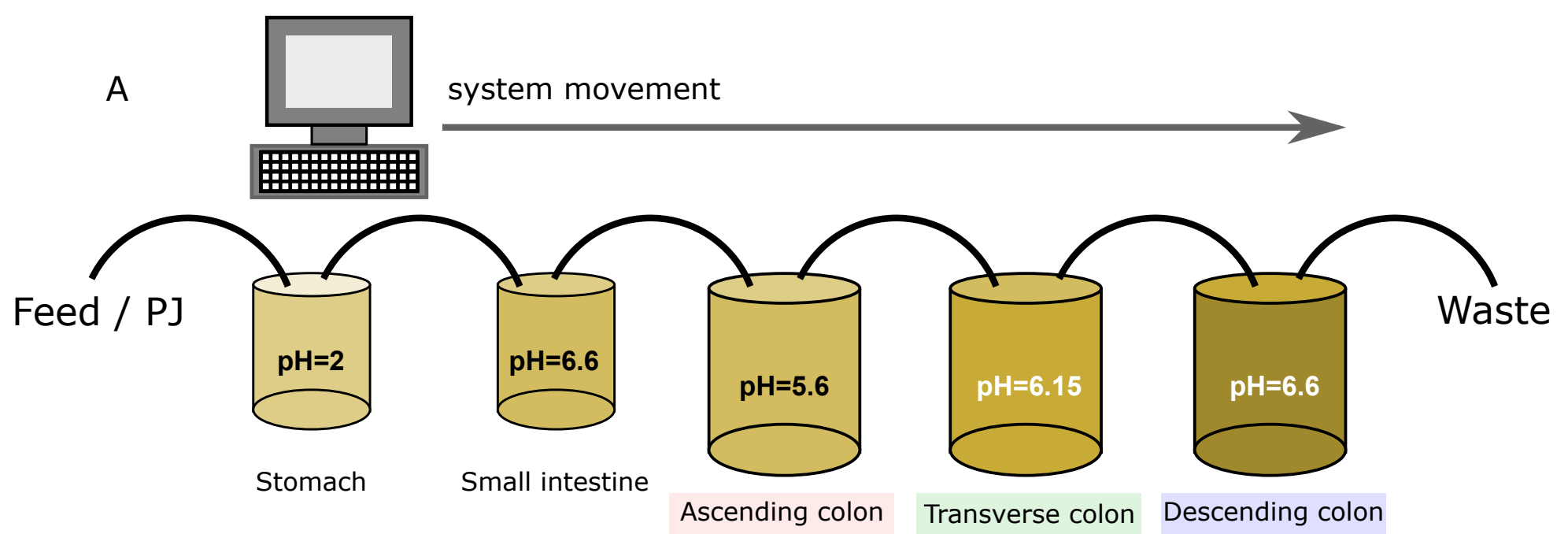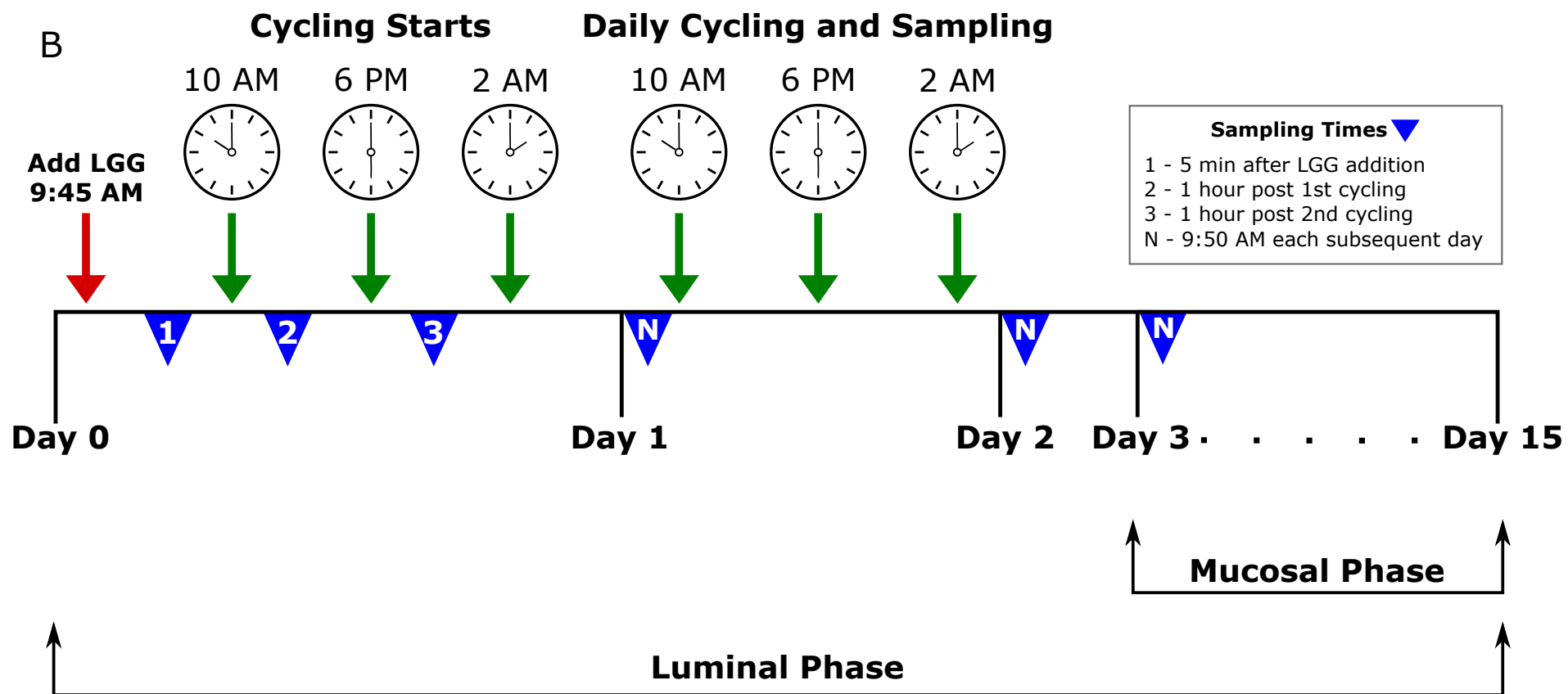

Supplement: Supplementary file 1 [file foods-12-02105-s001.zip › Figure S1.pdf]

Number of reads mapped

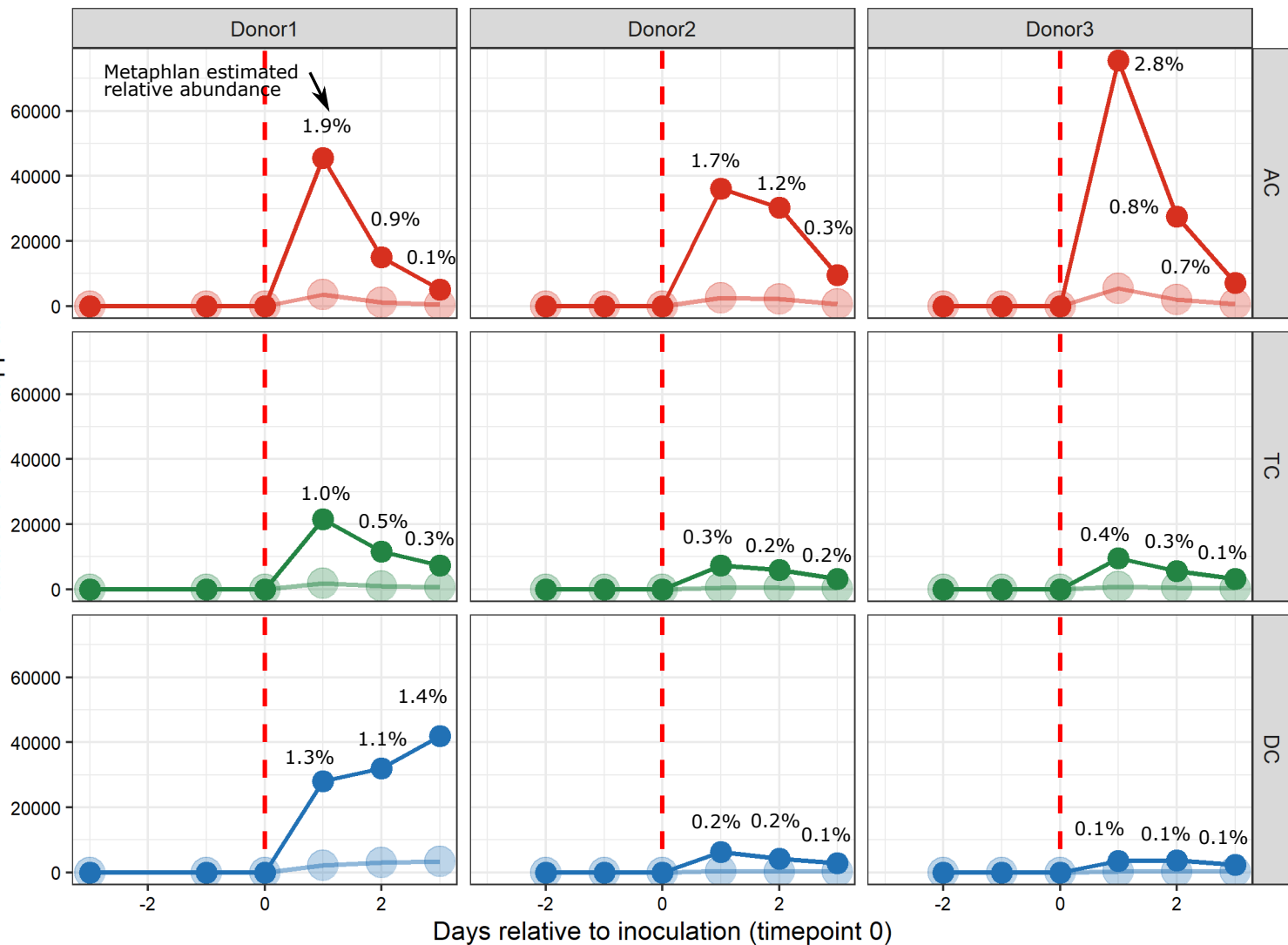

Supplement: Supplementary file 1 [file foods-12-02105-s001.zip › Figure S2.pdf]
